# Research on the design and application of “MOOC + flipped classroom” for basketball courses in colleges and universities from the perspective of education modernization

**DOI:** 10.3389/fpsyg.2023.1060257

**Published:** 2023-01-25

**Authors:** Ti Hu, Hong Liu, Fan Xia

**Affiliations:** ^1^College of P.E. and Sports, Beijing Normal University, Beijing, China; ^2^The High School Affiliated to Beijing Normal University, Beijing, China

**Keywords:** education modernization, “MOOC+ flipped classroom” teaching mode, college, basketball professional course, teaching design and application

## Abstract

**Background:**

The wave of education information based on the “Internet+” has swept the world, and the traditional teaching mode can no longer meet the new needs of education teaching, so the teaching mode of “MOOC+ flipped classroom” has attracted widespread attention educators.

**Objective:**

This study explores the effect of “MOOC+ flipped classroom” on the teaching design and application of basketball courses in colleges and universities from the perspective of education modernization in order to promote the development of students’ core literacy and provide a more theoretical basis and practical support for the in-depth research and promotion of “MOOC+ flipped classroom” teaching mode.

**Methods:**

This study adopts a quasi-experimental design to study the teaching of basketball courses in colleges and universities based on “MOOC +flipped classroom.” The experimental class adopted “MOOC+ flipped classroom” teaching (34 students), and the control group adopted traditional classroom teaching (30 students). Before and after the 16-week intervention, the student’s learning effects were measured by basketball skill level assessment, Cooperation Ability Scale for University Students, Utrecht Work Engagement Scale-student, and Self-regulated Learning Scale, and the data were analyzed by independent sample *t*-test and repeated-measures ANOVA.

**Conclusion:**

(1) Compared with the traditional classroom teaching mode, the “MOOC+ flipped Classroom” teaching mode is innovative in terms of teaching philosophy, teaching resources, and teaching methods, which promotes the change of education informatization and further promotes the realization of education modernization. (2) The “MOOC+ flipped classroom”-based teaching design for basketball courses benefits students’ *basketball skill level*, *study engagement*, *cooperation ability*, and *self-regulated learning ability*, thus effectively promoting the students’ core literacy.

## 1. Introduction

The essence of education modernization is the modernization of human development, which is the path to comprehensive human development (Chu, 2013). Education modernization, as an essential direction of educational reform and development in various countries in the global context, is supported by local, national, and international cooperation initiatives and involves different educational fields (Forma, 2016). The application of information technology is necessary for the educational modernization of processes (Oseredchuk et al., 2022). Information technology has a revolutionary impact on education, and its popularity and penetration will change the ecological environment for implementing effective education strategies and provide transformative ideas and challenges for implementing strategic education goals. Education informatization is core characteristic of modernization of education (Hu et al., 2011), and it is used information technology to stimulate the modernization of education, promote high-quality education development, and further realize the modernization of human beings in the new era (Guan, 2012).

How to introduce modern information technology in the education system and promote education modernization with the help of information technology is one of the problems to be solved today. Since the 21st century, the rapid development of the Internet and the emergence of new technologies and media have changed people’s habits and ways of working, and the wave of education informatization based on “Internet+” has swept the world (Zhao, 2020). Education is, therefore, in a new stage of profound informatization changes (Shi, 2021). Education informatization refers to the general application and promotion of information and information technology in education and teaching and the education and teaching sectors (He, 2011). It emphasizes the need to use modern technology to accelerate the reform of talent training mode and build a networked, digital, intelligent, personalized, and lifelong education system. The deep integration of information technology and education teaching is the core content of education informatization, and using information technology to innovate teaching modes and methods is an important way to promote deep integration. The traditional teaching mode can no longer meet the needs of modern education and teaching for talent development (Wen, 2022), but in higher education teaching practice, the traditional teaching mode centered on a classroom, teacher, and textbook is still more often used for teaching (González Velasco et al., 2021). There are problems such as the traditional teaching concept of taking teachers as the main body of teaching (Yang et al., 2021), lack of teaching resources (Niu et al., 2019), and single teaching method (Wu et al., 2021). Current higher education needs to explore new teaching models with the help of modern information technology, to promote innovation and change in education informatization, and further promote the realization of education modernization.

As a typical teaching model of reconstructing the learning process in information-based education, the flipped classroom changes the traditional roles of teachers and students. It redesigns classroom time by reversing knowledge transfer and knowledge internalization, thus stimulating students’ subjectivity and motivation, improving teaching efficiency, and truly realizing teaching to students’ abilities (Gan, 2018). The flipped classroom teaching model has unique advantages over traditional education models (Gong and Zhou, 2022). Its effectiveness in promoting academic achievement (O'Flaherty and Phillips, 2015; Akçayır and Akçayır, 2018; Wang and Zhu, 2019), learning attitude (Karadag and Keskin, 2017), and learning satisfaction (Gilboy et al., 2015; Kang and Kim, 2021), learning investment (Hung, 2015), and core literacy (Guo W. J. and Liu J. L., 2017; Cheng et al., 2020) has been verified in practice. Among them, the promotion effect for core literacy can be reflected by academic achievement, independent learning ability (Guo W. J. and Liu J. L., 2017), teamwork ability (Liu et al., 2022), and analytical and problem-solving ability (Cheng et al., 2020). The flipped classroom has to some extent, solved the problems of a single form of teaching in physical education courses, old teaching methods (Ge et al., 2018), and limited time for skill learning (Campos-Gutiérrez et al., 2021), and can better improve students’ attitude toward exercise, skill mastery (Wang, 2019) and motivation, and autonomy and interaction in the learning process (Hinojo Lucena et al., 2020), but there are also problems such as insufficient curriculum resources to be solved (Liu, 2015; Implementation Opinions of the Ministry of Education on the Construction of First-class Undergraduate Curriculum, 2019).

Flipped classroom provides a mature teaching model for education and teaching, but implementing flipped classroom needs quality teaching resources. Online open courses have brought about changes in learning styles, such as self-directed learning, fragmented learning, and learning without time and space constraints, which have injected new ideas of teaching and learning reform (Du et al., 2021). MOOC, in particular, is an innovative teaching and learning phenomenon rapidly penetrating higher education and has received wide attention from educators (Ahmed et al., 2022). It breaks the barrier between classroom teaching and practice teaching, realizing the comprehensive integration of different teaching links (Shu, 2018). In addition, it has gradually attacked traditional classroom teaching with its advantages of high-quality resources, rich teaching formats, convenient learning methods, and robust interactivity. However, problems such as low completion and high dropout rates of participants (Klemke et al., 2018) and the lack of direct teacher-student contact and effective interaction need to be addressed (Liu et al., 2018).

Based on the above, MOOC and flipped classroom form a complementary advantage. The pre-class learning under flipped classroom teaching can effectively play the advantages of MOOC course in terms of independent learning and personalized teaching and curb the problems of low course completion rate, lack of learning experience, and difficulty in assessing learning effects (Gao, 2014); while MOOC can provide high-quality pre-class learning resources for flipped classroom teaching, which in turn promote the effectiveness of classroom teaching. Therefore, this study takes the basketball course of college physical education majors as the entry point, innovates the teaching design of “MOOC+ flipped classroom,” and explores the benefits of this teaching mode to improve the core literacy of college students in order to promote the innovation and change of education informatization and the realization of education modernization.

## 2. Materials and methods

### 2.1. Study design

This study was conducted to study the teaching of basketball courses in colleges and universities based on “MOOC + flipped classroom.” A quasi-experimental design was adopted, and 64 students were grouped to form an experimental class (34 students) and a control class (30 students) with the physical education students of Beijing Normal University in the class of 2020 as the teaching subjects. The experimental class was taught in a “MOOC+ flipped classroom,” while the control class was taught in a traditional classroom. The teachers, content, schedule, and venue of the two classes are the same, and there are only differences in the teaching mode. The teaching time is 16 weeks, 64 h, 32 sessions, and 90 min per session.

It should be noted that the control class was taught in a traditional classroom format for 16 weeks, with only a few additional questionnaires in the first and 16th weeks. The students’ learning sequence was classroom learning, post-class review, completion of assignments, and assessment. Specific instruction was implemented as follows: (l) Learners began each module of basketball course content in an offline classroom; (2) During the class, the teacher explained basketball theory and explained and demonstrated practical basketball skills, and students engaged in passive learning and involved in some exercises and pair and group activities that match the interactive activities of the experimental class; (3) Each class lasted 90 min; (4) After the class, students conducted a self-directed review, practice, and complete post-class assignments; (5) A short class quiz at the beginning of the next session to assess mastery of theory and practical skills learned in the previous session through in-class or post-class study; and (6) A theory and practice assessment at the end of the 16-week semester.

### 2.2. “MOOC+ flipped classroom” based teaching design for basketball courses in colleges and universities

#### 2.2.1. Theoretical basis

Along with the profound changes in education modernization, information-based teaching is the product of combining theory, technology, and practice. In addition to the efforts in technology and practice, it is necessary to be familiar with and reasonably apply the theories related to information-based teaching. In this study, the *constructivist learning theory*, *mastery learning theory*, *cognitive load theory*, and this model are combined organically and throughout the teaching process to ensure the necessary conditions for the scientific feasibility of this teaching model. Specifically, teacher view, student view, teaching view, and learning view of *constructivist learning theory* provide a new theoretical framework of the teaching model and fully reflect the concept of “teachers leading and students as the main body “(Ausubel, 1978; Guo X. and Liu L., 2017); *Mastery learning theory* focuses on individual student differences and provides new ways of learning for this teaching model, broadening the time limits of learning by providing the personalized help needed as well as the extra study time required, which helps students to learn more effectively and master the content better (Bloom, 1976, 1979). *Cognitive load theory* provides new theoretical guidance for the effective implementation of this instructional model by regulating the reduction of learners’ external cognitive load, optimizing the associated cognitive load, and regulating the internal cognitive load (Paas and Van Merriënboer, 1994; Pollock et al., 2002; Onah et al., 2021).

#### 2.2.2. Preparation stage

##### 2.2.2.1. Teaching objectives

The development of information technology is causing a revolution in education, which makes the education ecology change, the learning environment is changing, the learning content is changing, the learning means are changing, and the teacher-student relationship is changing” (Gu, 2018), the traditional teaching single teaching goal can hardly meet the needs of today’s teaching. This teaching design achieves the overall teaching objectives by reaching three stages: before, in, and after class. In the before class phase, students acquire theoretical knowledge related to teaching basketball skills and tactics (connotations, movement essentials, critical points, easy mistakes, etc.) and then initially establish a visual representation by combining the organized practice forms and peer-to-peer learning and practice in the class. In class stage, through targeted error correction, guidance, and practice, to solve students’ questions and correct technical movements to shorten the time to explain the critical content. Moreover, through the situation, collaboration, dialogue, competition, and other organizations, fully mobilize students’ learning enthusiasm so that students to internalize and consolidate what they have learned in the process of high-density “learning,” “practice,” and “competition” scenarios. After class stage, students complete the post-course tasks together through self-practice and practical application to enhance their practical ability and professionalism in using skills; thus, students can systematically master the basic theory, fundamental skills, and basic methods of professionalism in physical education (Huang et al., 2016).

##### 2.2.2.2. Teaching content

The basketball course is a compulsory course for physical education students. According to the syllabus and teaching design arrangement, the teaching content is divided into theory and practice. The theoretical part includes the technical and tactical principles of basketball skills, theories and methods of teaching and training; the practical part is mainly physical activities, including technical and tactical learning and teaching practice. In this teaching mode, teachers deeply analyze the structural relationship of basketball teaching content, combine teaching objectives and learners’ characteristics, screen out the knowledge of sports skills that students need to master at different stages, and integrate the ideological elements such as solidarity and cooperation, responsibility, and commitment into the whole process of basketball teaching appropriately. Through scientific and reasonable class time arrangement, taking into account the penetration and integration of theoretical knowledge and teaching practice, the learning effect of basketball knowledge and skills is improved comprehensively.

##### 2.2.2.3. Teaching platform

The information age, characterized by big data and the “Internet +,” has deepened the connotation of education modernization (Shi, 2021). In this study, we used the developed “Basketball—Basic Techniques” MU course, using the information technology network platform—China University MOOC (Li and Hu, 2018), which provides a wide range of course information resources. At the same time, in order to play a more efficient role in the online teaching of professional courses and to facilitate professional teaching and management, small-scale restricted online teaching is conducted through Small Private Online Course (SPOC, Called “post-MOOC”). SPOC and MOOC both use the Internet for learning. However, the SPOC audience is limited to a particular class, primarily used within the school, and easier to manage and monitor the information learning of users than MOOC. We thoroughly use SPOC and course WeChat groups as channels for teacher-student and student–student communication and interaction to monitor and give real-time feedback on students’ learning. It aims to provide online openness and real-time interaction for students to learn anytime, anywhere, selectively, and purposefully, without the constraints of geography, time, and space. Through various functional sections such as questions, discussions and answers, quizzes, and assignments, theoretical and practical exams, and course evaluation, we can realize “fine” and “refinement” of teaching resources and “individualization” and “personalization” of the teaching process, this is meet students’ needs better.

##### 2.2.2.4. Teaching evaluation

The modernization of the teaching evaluation system is the key to education. Only by establishing a concrete and scientific index system can we measure and test the standard of the implementation level of educational modernization (Shi, 2008). In order to scientifically and effectively evaluate the teaching effectiveness of this teaching design, a diagnostic, formative, and summative trinity teaching evaluation system is established based on the teaching objectives at different stages, and a comprehensive, integrated, scientific, and objective evaluation of the learning effect and teaching quality from different stages, aspects, and ways. The diagnostic evaluation is conducted before the semester course to understand students’ basic mastery level through a unified theory and skill test. Moreover, the class test begins before the new lecture by asking random questions and skill demonstrations to test their readiness for learning the newly taught knowledge. Formative assessment is the evaluation of students before, during, and after class. Before class stage, formulate corresponding learning tasks or test questions according to the important and difficult points of teaching and collect students’ feedback; In class stage, group competition, team display, or mutual evaluation between teachers and students are interspersed for evaluation; After class stage, teachers’ evaluation and mutual evaluation are adopted to supervise students’ after-school learning and complete the comprehensive evaluation of students’ learning effect.

Summative evaluation tests by students’ theoretical knowledge and motor skills at the end of the semester and forms a comprehensive evaluation of the course, and combining MOOC platform learning data (video clicks, forum replies, online tests, etc.) and evaluation results before, in, and after class. While helping students analyze and correct their shortcomings, it also provides reference and a basis for improving later course teaching.

#### 2.2.3. Teaching implementation phase

**Figure 1:**
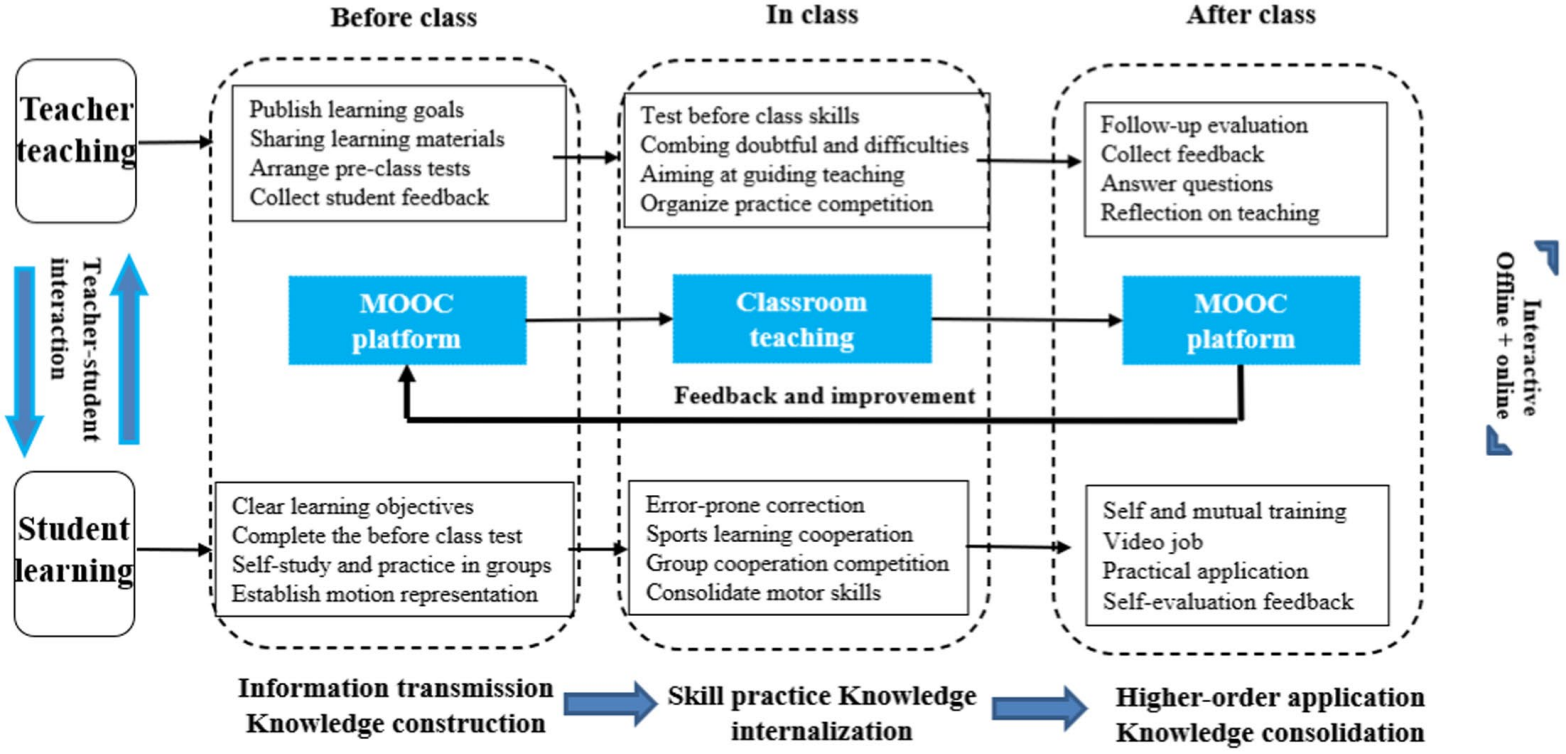
Teaching design of “MOOC+ flipped classroom” for basketball courses in colleges and universities.

This instructional design is divided into before class knowledge transfer stage, in class knowledge internalization stage, and after class knowledge consolidation stage (Figure 1). Mainly from the perspective of teachers and students, the three stages of teaching activities are elaborated and analyzed in detail so that students can achieve the stage goals of knowledge construction, skill consolidation, and high-level application (Hu et al., 2022).

##### 2.2.3.1. Before class knowledge transfer phase

Before class stage is the key stage of this teaching model, and the learning effect in this stage directly affects the learning starting point in class (Zhao, 2014). Only when a certain learning effect is achieved in the pre-class stage can the mastery of knowledge and skills be further improved in class.

In terms of learning objectives, teachers need to clarify the learning objectives to avoid the blindness of students’ independent learning, increase the learning load, and facilitate the clarification and mastery of the essential and challenging points of the learning content. From the perspective of theoretical knowledge, teachers should master the theoretical knowledge related to technical and tactical teaching, such as movement methods, tactics, rules, etc.; from the perspective of movement skills, teachers should master the cognitive imitation of techniques and tactics before class (Li and Wang, 2015). In terms of learning materials, teachers divide the overall requirements of teaching objectives and knowledge system into several knowledge points according to this course’s characteristics, fragmenting the course’s content. Select courses in the MOOC platform around the fragmented knowledge, guide students to selectively enroll in classes, assign learning tasks, and ask thinking questions, students’ completion can be recorded to facilitate teachers’ classroom tasks or projects that can be managed and evaluated by the instructor. Among them, for skills teaching, intuitive pictures, and videos can be used to explain and reduce learners’ external cognitive load; For the study of theoretical knowledge, text summary or courseware and other text resources can be used in the video to assist learning, to help students achieve the pre-class teaching objectives and enrich students’ relevant knowledge reserves. Furthermore, it can reduce the internal cognitive load of learning in class and initially establish visual representation. Then, peers help each other to learn and practice, perceive muscle proprioception, and establish kinesthetic representation during the practice. In pre-class learning, problems that are difficult to solve can be communicated in groups or discussed with teachers and classmates on the MOOC platform to construct personal knowledge (Zeng et al., 2020).

##### 2.2.3.2. In class knowledge internalization stage

In class phase is the process of solving students’ queries, correcting technical movements, and reinforcing exercises, and the teaching process in which teachers use elements, such as situations, collaboration, dialogues, and competitions to organize and guide students to solve problems and complete the construction of knowledge (Chen and Zhou, 2017). Through the pre-course phase, students initially master the basic knowledge and skills. In the in-class stage, students further internalize their knowledge and skills through more targeted explanations, longer practice time, and higher practice density to achieve a more profound mastery of the learning content.

In class stage, teachers first answer students’ questions about the online test and MOOC platform before class learning. If most students have difficulties mastering the content, the teacher still explains the actual content (Wang, 2016); if most students have good mastery, the teacher organizes the next learning session. The teacher selects students by name or voluntarily to demonstrate the technique, and the teacher reviews, instruct and demonstrates the correct movement so that students can further establish a correct image of the movement and understand the various aspects of the movement. Subsequently, group practice and cooperative inquiry are conducted to give full play to students’ autonomy and motivation and improve classroom participation; Simultaneously, the teacher conducts roving instruction, carries out targeted learning methods, gives students targeted guidance, strengthens correct muscle memory, and eliminates incorrect technical movements. Then the teacher organization inter-group demonstrations and competitions to enable students to grasp the learned movements fully. Finally, the teacher summarizes the class.

Based on self-study before class, teaching can be more targeted in the class to sort out the learning content, shorten the time to explain the actual content, and give more class time to students to improve learning efficiency. In the process of high-density “learning” and “practice,” the content learned is continuously internalized and consolidated.

##### 2.2.3.3. After class knowledge consolidation stage

The main goal of after class stage is to evaluate and test what has been learned and to help students consolidate and improve the comprehensive application of skills (Zhen and Lu, 2017). At this stage, teachers can release post-class self-study assignments through the online platform and set student group assignments based on the problems in the classroom learning so that students can conduct a self-study and mutual learning. Provide students with answers to questions and discussion interactions in the SPOC online learning community, and complete the tracking and evaluation of students’ learning effects in this process. Such an online interactive environment generates and consolidates students’ mastery of techniques, increases inquiry skills, and promotes student development (Sun and Li, 2018).

Students’ integrated application of techniques and tactics requires, on the one hand, learning materials and practice experience in class, and on the other hand, much self-practice and practical application after class to achieve proficiency. The advantages of this teaching design can make students repeatedly watch the learning materials before class and combine them with the practice experience in class to understand better and remember and apply. In post-class practice, students can communicate with teachers and classmates through the SPOC platform promptly, and the after class feedback provides reference and guidance for later teaching to achieve compelling mastery and comprehensive application of knowledge and skills.

### 2.3. Data collection

#### 2.3.1. Measurement of cooperation ability

The *Cooperation Ability Scale for University Students* was developed by scholar Li et al. (2013). The scale is applicable to assess students’ *cooperative ability* and contains 43 items, consisting of two parts: collective awareness (cognitive) and cooperative skills. Collective awareness includes cooperative cognition, cooperative emotion, and cooperative intention, and cooperative skills include interpersonal support, conflict management, emotion regulation, and organizational leadership. The internal reliability consistency of the total scale was 0.94, and the split-half reliability was 0.87. The validity coefficients of each factor and the total questionnaire ranged from 0.64 to 0.83, and the validity coefficients of each factor ranged from 0.27 to 0.64. The scale’s reliability and validity and the structural model’s fit indicators all reached acceptable standards.

#### 2.3.2. Measurement of basketball skill level

The classroom teachers administered the subjective items of *basketball skill level* test content (half-court dribbling technique, free throw); two teachers administered the objective items (marching pass and catch, game), and the data were collected through the test results.

#### 2.3.3. Measurement of study engagement

The *Utrecht Work Engagement Scale-student* obtains from the translation of the *UWES-S scale* and the *Learning Performance Scale* by Chinese scholars, Professors Fang et al. (2008), and the reliability test. The internal reliability consistency of the scale is in the range of 0.82–0.95, the correlation coefficient is significant and in the range of 0.76–0.77, and the item loadings are in the range of 0.42–0.81, with good fit indicators and good reliability, which adopted for relevant studies.

#### 2.3.4. Measurement of self-regulated learning

The *Self-regulated Learning Scale* for College Students in Physical Education was developed by Chinese scholars Wu (2010). The results of its validation factor analysis showed that the RMSEA values reached the excellent standard, and the AGIF, CIF, IIF, NFI, and NNF work indices were all ≥0.90. The Cronbach coefficients of each subscale were above 0.78, and the full scale was 0.892. The results of the retest reliability analysis showed that the reliability coefficients of each subscale are 0.872, 0.821, 0.847, and 0.813, respectively, and the full scale is 0.913. It has high validity and reliability.

### 2.4. Statistical analysis

The data were analyzed using spss26.0 statistical software with a significance level of *p* < 0.05. In order to explore the influence of basketball courses on students under different modes, the measurement data of *basketball skill level*, *study engagement*, *cooperation ability*，and *self-regulated learning ability* were expressed as mean and standard deviation, and the data conformed to the normal distribution, with independent samples *t*-test for the pre-test data and repeated-measures ANOVA for the post-test data.

In addition, this study conducted a *post hoc* power analysis using the software G^*^Power (version 3.1.9.2; Kiel University, Kiel, Germany) to confirm the sample sizes. The power analysis with Effect size *f* = 0.2826 or 1.8348 (*partial η^2^* = 0.074 or 0.771), α error of probability = 0.05, total sample size = 64, Number of groups = 2, Number of measurements = 2, and correlation = 0.554. Within the chosen sample size, the power (1−β) is 0.99 or 1.00.

## 3. Results

### 3.1. Analysis of the pre-test results of basketball course design based on “MOOC +flipped classroom” teaching in colleges and universities

The results of the independent samples *t*-test on the pre-test result in the students in the experimental and control classes. The results showed (Table 1) that in terms of *basketball skill level* (*p* = 0.308 > 0.05), *study engagement* (*p* = 0.822 > 0.05), *cooperation ability* (*p* = 0.935 > 0.05), and *self-regulated learning ability* (*p* = 0.497 > 0.05), all *p* > 0.05. There was no statistically significant difference between the pre-test results of the experimental and control groups, and the students in the experimental and control classes were at the same level.

**Table 1:** Analysis of *t*-test results of independent samples in the experimental group and the control group.

	Group (*M* ± *SD*)		*t*	*p*
	Control group (*n* = 30)	Experimental group (*n* = 34)		
Basketball skill level	13.47 ± 4.18	12.44 ± 3.81	1,028	0.308
Study engagement	5.35 ± 1.23	5.41 ± 0.95	−0.226	0.822
Cooperation ability	3.61 ± 0.50	3.62 ± 0.61	−0.082	0.935
Self-regulated learning ability	3.87 ± 0.51	3.80 ± 0.39	0.683	0.497

### 3.2. Analysis of post-test results of basketball course design based on “MOOC+ flipped classroom” teaching in colleges and universities

A repeated measures ANOVA was performed on the data onto students in the experimental and control classes, and the results are shown in Table 2.

**Table 2:** Repeated measures ANOVA results of experimental group and control group.

	The source	Post-test		*F*	*p*
		Experimental group (*M* ± *SD*)	Control group (*M* ± *SD*)		
Basketball skill level	Time	20.29 ± 1.91	18.29 ± 1.92	209.150	0.000^**^
	Group			0.557	0.458
	Time ^*^ Group			11.878	0.001^**^
Study engagement	Time	5.82 ± 0.85	5.24 ± 1.17	1.905	0.172
	Group			1.818	0.182
	Time ^*^ Group			5.970	0.017^*^
Cooperation ability	Time	4.28 ± 0.41	3.86 ± 0.50	92.894	0.000^**^
	Group			3.668	0.060
	Time ^*^ Group			15.849	0.000^**^
Self-regulated learning ability	Time	4.38 ± 0.43	3.92 ± 0.54	13.856	0.000^**^
	Group			5.891	0.018^*^
	Time ^*^ Group			9.440	0.003^**^

^**^ means *p* < 0.01, ^*^means *p* < 0.05.

**Figure 2:**
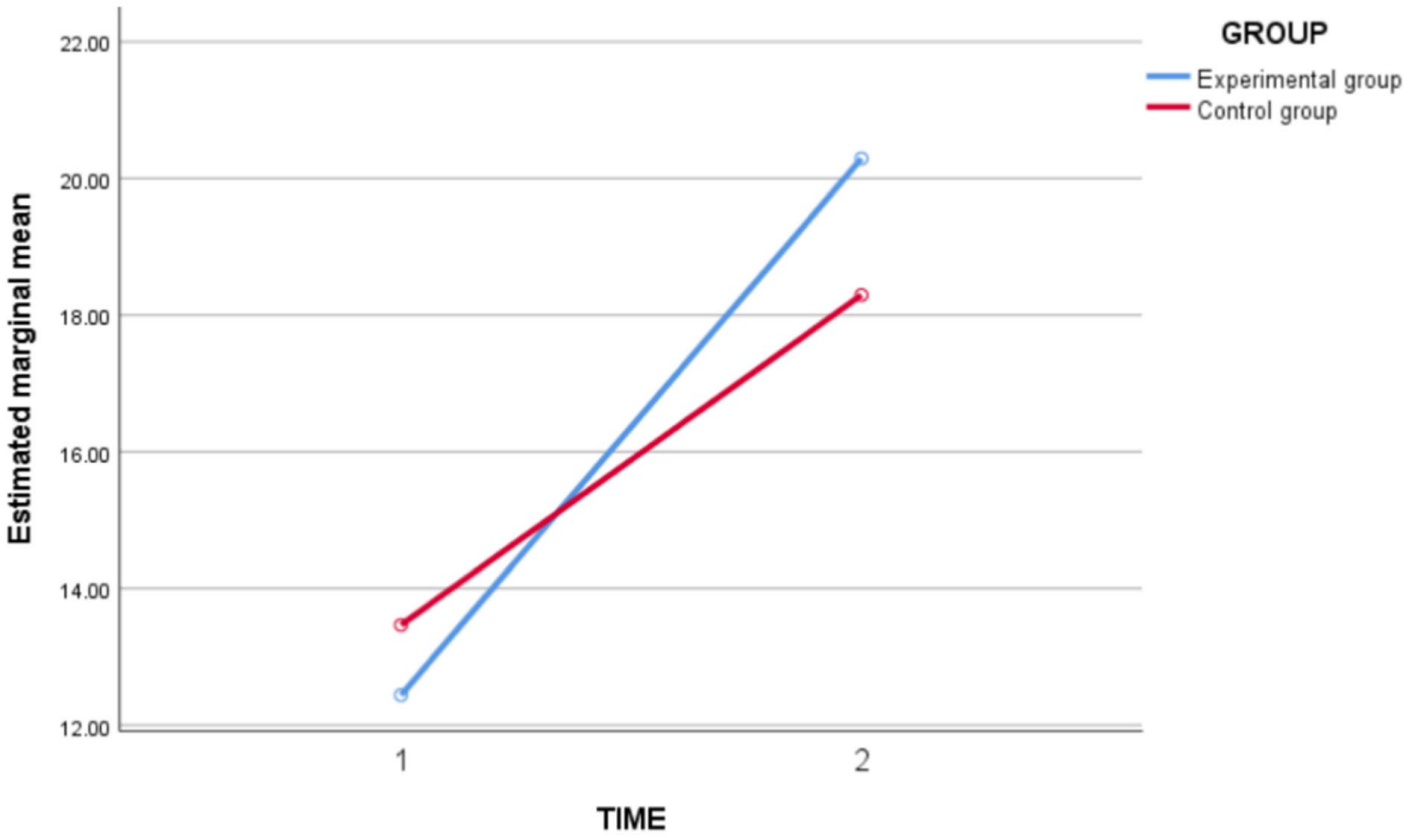
Schematic diagram of the changes in basketball skill level between the experimental and control groups before and after the intervention.

In terms of *basketball skill level* (Figure 2), the results of the repeated measures ANOVA showed a highly significant main effect of time [*F*_(1,62)_ = 209.150, *p* = 0.000 < 0.05, *partial η^2^* = 0.771], indicating that there was a highly significant difference in *basketball skill level* over time after instruction, and all in the direction of improvement. Time ^*^ Group interaction effect was highly significant [*F*_(1,62)_ = 11.878, *p* = 0.001 < 0.05, *partial η^2^* = 0.161], indicating that the experimental and control groups differed in the magnitude of change in basic *basketball skill level* before and after instruction. The between-group effect was not significant [*F*_(1,62)_ = 0.557, *p* = 0.458 > 0.05, *partial η^2^* = 0.009]. Then, a group simple effects analysis was carried out, and the results showed (Table 3) that there was no significant difference in the basic *basketball skill level* between the experimental and control groups before the intervention (*p* = 0.193 > 0.05). After the intervention, there was a significant difference in the post-test basic *basketball skill level* between the experimental and control groups (*p* = 0.012 < 0.05), indicating that the post-test basic basketball skill level in the experimental group was significantly greater than the post-test basic *basketball skill level* of the experimental group was significantly greater than that of the control group.

**Table 3:** Simple effects analysis of basketball skill level group between experimental group and control group before and after the intervention.

Time	Group	Average difference	Standard error	*t*	*p*
Pre-test	Control group—Experimental group	1.026	0.783	1,310	0.193
Post-test	Control group—Experimental group	−1.995	0.783	−2,547	0.012^*^

^*^means *p* < 0.05.

In conclusion, the experimental class improved the *basketball skill level* of students and was better than the control group.

**Figure 3:**
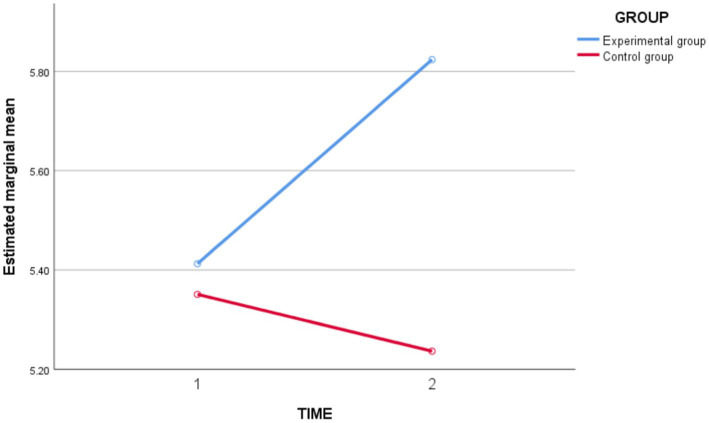
Schematic diagram of changes in study engagement on the experimental group and the control group before and after the intervention.

In terms of *study engagement* (Figure 3), the results of the repeated measures ANOVA showed that the main effect of time were not significant [*F*_(1,62)_ = 1.905, *p* = 0.172 > 0.05, *partial η^2^* = 0.030]. The time ^*^ group interaction effect was significant [*F*_(1,62)_ = 5.970, *p* = 0.017 < 0.05, *partial η^2^* = 0.088], indicating that the magnitude of change in student *study engagement* before and after instruction differed from the experimental and control groups. The group effect was not significant [*F*_(1,62)_ = 1.818, *p* = 0.182 > 0.05, *partial η^2^* = 0.028]. Then, a group simple effects analysis was carried out, and the results showed (Table 4) that before the intervention, there were no significant difference in *study engagement* to between the experimental and control groups (*p* = 0.815 > 0.05). After the intervention, there was a significant difference in the post-test *study engagement* level between the experimental and control groups (*p* = 0.029 < 0.05), and the post-test *study engagement* level of the experimental group was significantly greater than the post-test *study engagement* to the control group level score.

**Table 4:** Simple effects analysis of study engagement group between experimental group and control group before and after intervention.

Time	Group	Average difference	Standard error	*t*	*p*
Pre-test	Control group—Experimental group	−0.062	0.264	−0.235	0.815
Post-test	Control group—Experimental group	−0.587	0.264	−2,227	0.029^*^

^*^ means *p* < 0.05.

In conclusion, the experimental class improved the students’ *study engagement* and was better than the control group.

**Figure 4:**
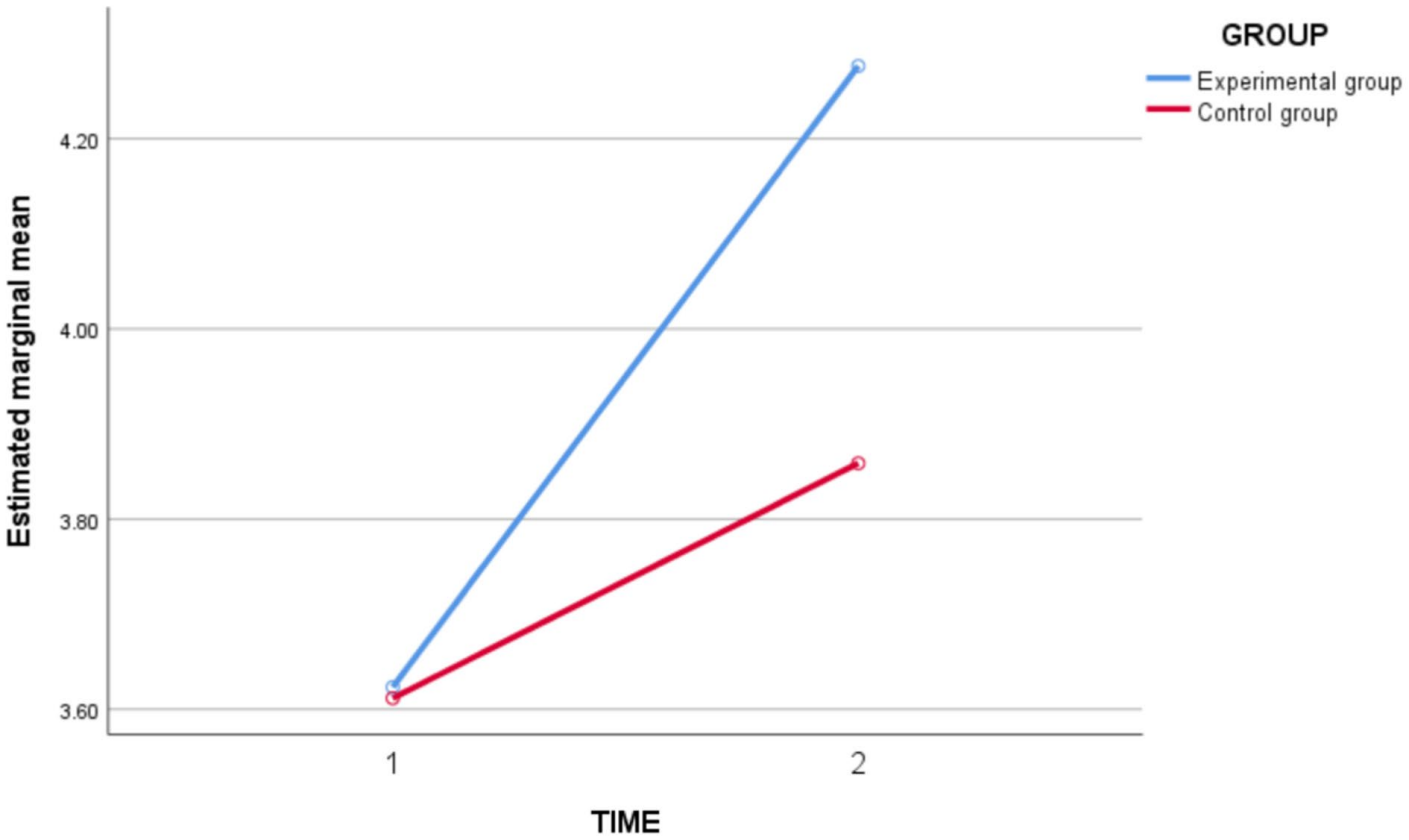
Schematic diagram of the changes in cooperation ability between the experimental group and the control group before and after the intervention.

In terms of *cooperation ability* (Figure 4), the results of the repeated measures ANOVA showed a highly significant main effect of time [*F*_(1,62)_ = 89.256, *p* = 0.000 < 0.05, *partial η^2^* = 0.590], indicating a highly significant difference in *cooperation ability* over time, and both in the direction of improvement. Time ^*^ Group interaction effect was significant [*F*_(1,62)_ = 18.175, *p* = 0.000 < 0.05, *partial η^2^* = 0.227], indicating that the experimental and control groups differed in the magnitude of change in the level of c*ooperation ability* before and after instruction. The between-group effect was not significant [*F*_(1,62)_ = 3.269, *p* = 0.075 > 0.05, *partial η^2^* = 0.050]. Then, a group Simple effects analysis was carried out, and the results showed (Table 5) that before the intervention, there was no significant difference in the level of *cooperation ability* between the experimental and control groups (*p* = 0.928 > 0.05). After the intervention, there was a significant difference in the level of post-test *cooperation ability* between the experimental and control groups (*p* = 0.002 < 0.05), and the level of post-test *cooperative ability* in the experimental group was significantly greater than in the post-test basketball. The basic technical level score of the experimental group was significantly greater than that of the control group.

**Table 5:** Simple effects analysis of cooperation ability group between experimental group and control group before and after intervention.

Time	Group	Average difference	Standard error	*t*	*p*
Pre-test	Control group—Experimental group	−0.012	0.128	−0.090	0.928
Post-test	Control group—Experimental group	−0.418	0.128	−3,265	0.002^**^

^**^ means *p* < 0.01.

In conclusion, the experimental class improved the students’ *cooperation ability* and was better than the control class.

**Figure 5:**
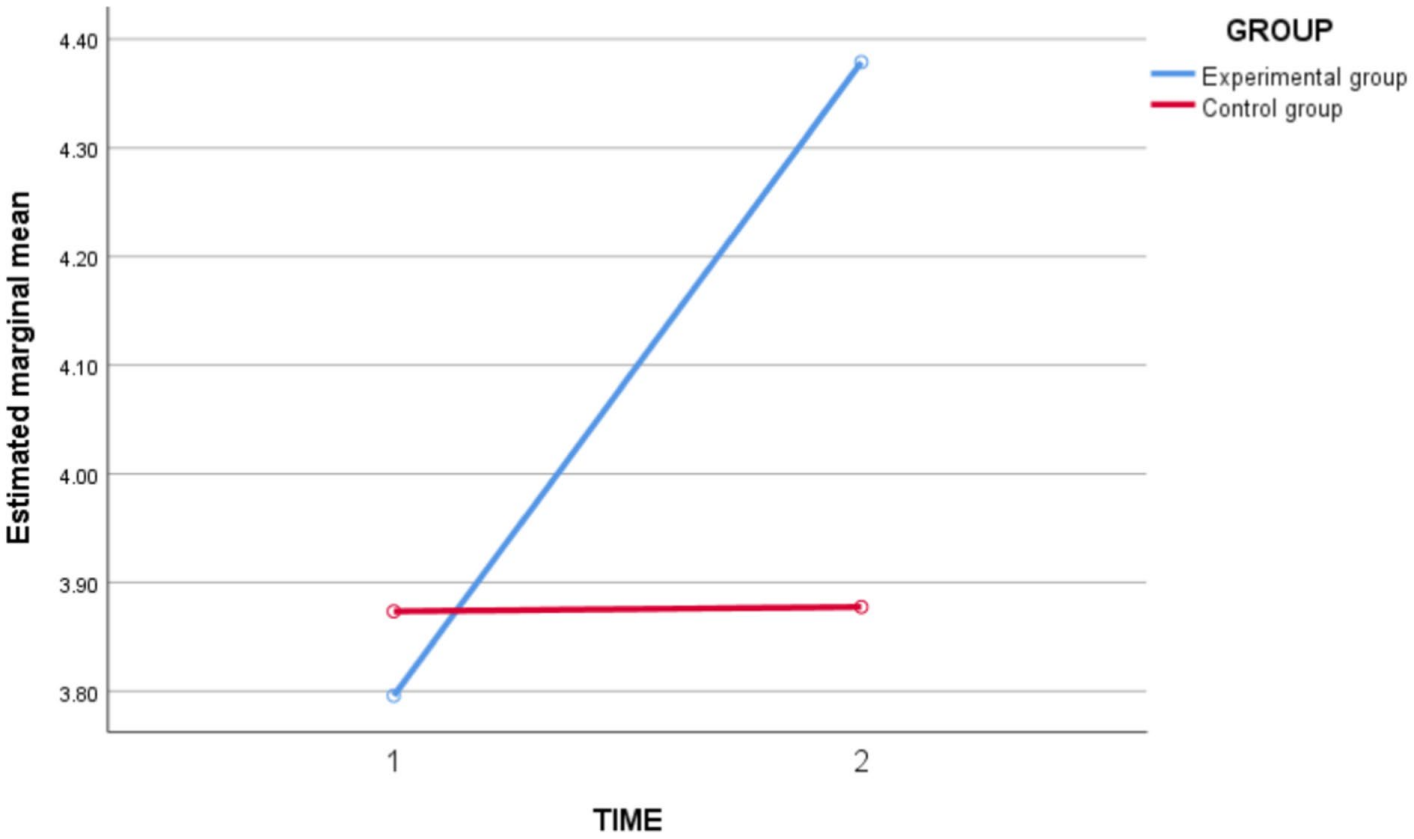
Schematic diagram of changes in self-regulated learning ability between the experimental group and the control group before and after the intervention.

In terms of *self-regulated learning ability* (Figure 5), the results of the repeated measures ANOVA showed a highly significant time main effect [*F*_(1,62)_ = 22.650, *p* = 0.000 < 0.05, *partial η^2^* = 0.268], indicating a tendency toward the level of *self-regulated learning ability* to change into time. Time ^*^ Group interaction effect was highly significant [*F*_(1,62)_ = 22.037, *p* = 0.000 < 0.05, *partial η^2^* = 0.262], indicating that the experimental and control groups differed in the magnitude of change in the level of *self-regulated learning ability* before and after instruction. The between-group effect was significant [*F*_(1,62)_ = 4.930, *p* = 0.030 < 0.05, *partial η^2^* = 0.074], indicating that there was a significant difference in the level of independent learning between the experimental and control groups.

In conclusion, the experimental class improved the students’ *self-regulated learning ability* and was better than the control group.

## 4. Discussion

In exploring the teaching design of basketball courses in colleges and universities based on “MOOC + flipped classroom.” Compared with the traditional classroom teaching mode, the “MOOC + flipped classroom” teaching mode is innovative in terms of teaching concepts, teaching resources, and teaching methods, thus promoting the change of education informatization and the realization of education modernization.

In terms of teaching philosophy, the “MOOC + flipped classroom” teaching model emphasizes that the classroom is organized around the learner, and the student is the main subject and the center of the classroom, reflecting a modernized teaching philosophy of “learning before teaching” emphasized by the modernization of education (Li C. et al., 2020). The emergence of the “MOOC+ flipped classroom” teaching mode gives full play to learners’ initiative and learning enthusiasm, conforms to learners’ cognitive habits, and optimizes the learning effect of teaching and learning. However, the traditional classroom adopts the “transfer-receive” teaching mode, which reflects the teaching concept of “teaching first and learning later” (He, 2014), in which the teacher is at the center of the classroom and students are always in the passive state of following the teacher’s ideas. The “MOOC+ flipped classroom” teaching concept is undoubtedly an innovation of the traditional classroom teaching concept based on the modernization of education.

In terms of teaching resources, the MOOC platform brings together high-quality teaching resources and teaching materials of famous global teachers’ good courses. It eliminates the boundaries of time and space, breaks the monopoly of famous traditional schools on teaching resources, and has rich teaching content and strong independent selectivity of students (Wang, 2017). The teaching resources of the traditional classroom mainly come from a textbook, reference books, and the knowledge possessed by the teacher. MOOC innovates on the limited teaching resources of traditional classrooms with the help of information technology.

In terms of teaching methods, when teaching online in the “MOOC + flipped classroom” mode, teachers publish course materials and assign pre-reading and review tasks, pose reflection questions, exchange questions and doubts with students on the online platform, and assign exercises and tests after class. The teacher used teaching methods, such as independent learning, task-driven, discussion, and practice methods; When teaching offline, the teacher carried out activities such as explaining, demonstrating to solve students’ queries and correcting technical movements, organizing intra-group collaboration and inter-group competitions to strengthen skills, and group demonstrations. The teacher used teaching methods, such as lectures, demonstrations, discussions, competitions, and presentations. However, the traditional classroom teaching method is that the teacher instills knowledge into students through lectures and demonstrations, and students passively accept and repeat the exercises to achieve skill proficiency; After class, students review and complete after-class assignments on their own. Compared with the single teaching method under traditional classroom teaching, the “MOOC + flipped classroom” teaching mode provides a variety of teaching methods for students according to the teaching reality and specific teaching content, which genuinely realizes the teaching according to the material.

In exploring the teaching effectiveness of “MOOC + flipped classroom” college basketball course teaching design for physical education students in practice. These results show that after one semester of teaching, “MOOC+ flipped Classroom” improved students’ *basketball skill level*, *study engagement*, *cooperation ability*, and *self-regulated learning ability*, and was better than the control group.

The “MOOC+ flipped classroom” helped improve students’ basketball skill level and was better than traditional teaching. Related studies have shown that “MOOC+ flipped classroom” teaching can help improve students’ performance in practical courses (Tang et al., 2018; Wang et al., 2018), and students are more proficient in physical skills with flipped classroom teaching in college sports (Zhao, 2022). Moreover, flipped classroom teaching in college sports basketball classes can improve students’ professional basketball skills, which is consistent with the results of this study (Wang, 2021). The reason for this is that traditional teaching is based on one-way transmission and faces the limitation of insufficient class time, while “MOOC+ flipped classroom” provides new ideas and methods. Students can study independently through the MOOC platform before the class, lock the important and difficult points of the course, and access the online course freely, which helps them understand and prepare for the course. Secondly, students majoring in physical education have a particular foundation, and the advanced study of online courses helps to develop skills (Wang and Chen, 2022). Meanwhile, students can adjust their learning progress according to the rhythm, watch specific topics repeatedly for deeper understanding (Li W. J. et al., 2020; Zhao, 2022), and improve their analysis and problem-solving ability (Jia, 2020). Moreover, the set of homework at the end of the class is conducive to the complete consolidation of students’ skills so that students can fully reflect on themselves, know themselves, evaluate themselves, and make immediate changes in their learning concepts (Cheng et al., 2020), thus effectively improving students’ *basketball skill level* and promoting the development of their core literacy.

“MOOC + flipped classroom” teaching helps to improve students’ *study engagement* and is better than traditional teaching. The study showed that the MOOC+ flipped classroom teaching mode helps to improve students’ interest and study engagement (Tang et al., 2018), and Hu ‘s study also obtained the same results (Hu, 2021). In this study, it is believed that under the “MOOC+ flipped Classroom” teaching model, the teacher distributes the classroom led to the students, and the students conduct self-study and group study according to the tasks assigned by the teacher, and the classroom group study and group competition. The assignment to teaching tasks can clarify students’ learning goals and motivation, and strong motivation can stimulate students’ self-confidence and energy so that their emotional identification and inner cognition can be deepened, which promotes students’ *study* engagement. The atmosphere of positive student *study engagement* is conducive to student’s increased self-efficacy, which promotes lifelong physical education and is more suitable for cultivating students’ core literacy (Ma, 2020).

“MOOC + flipped classroom” teaching helps to improve students’ *self-regulated learning ability* and *cooperation ability* and is better than traditional teaching. Studies have shown that with MOOC and flipped classroom teaching mode, students change from passive to active in the flipped classroom teaching mode and develop student innovation and autonomy (Fan and Meng, 2019). In surgical nursing courses, MOOC-based flipped classroom teaching is more helpful in improving teamwork skills than traditional teaching. In addition, some studies further illustrate that the design of learning before class, learning in class through group discussion and collaboration, improving competency after class, and giving evaluation feedback makes full use of the advantages of MOOC and flipped classrooms to develop students’ *self-regulated learning ability* and *cooperative ability* (Wang, 2018). Investigate its reasons, On the one hand, the pre-class assignments require students to complete independently, and the teacher provides “fragmented” knowledge for students’ independent learning through the MOOC teaching platform, while the post-class assignments are set to fully stimulate students’ independent learning. On the other hand, the group study before class, from problem identification to problem-solving, exercises students’ *cooperation ability*; the group practice, cooperative investigation, and group competition during class make students feel the power of teamwork; after class, the setting of group after-class homework and the discussion and interaction in MOOC online learning community fully stimulate students’ cooperative learning ability. Self-directed learning and cooperative learning shape students’ ability to personalize learning and cultivate information awareness, social responsibility, diligent reflection, joyful learning, good learning, and other core literacies, which promote the improvement of students’ core literacy (Cheng et al., 2020; Wang and Chen, 2022).

## 5. Conclusion

(1) Compared with the traditional classroom teaching mode, the “MOOC+ flipped Classroom” teaching mode is innovative in terms of teaching philosophy, teaching resources, and teaching methods, which promotes the change of education informatization and further promotes the realization of education modernization. (2) The “MOOC+ flipped classroom”-based teaching design for basketball courses benefits students’ *basketball skill level*, *study engagement*, *cooperation ability*, and *self-regulated learning ability*, thus effectively promoting the students’ core literacy.

## 6. Suggestion

In future teaching research, “MOOC + flipped classroom “can be applied to the teaching of different sports, so that it can better cultivate students ‘core literacy skills and effectively promote their overall development. At the same time, physical education teachers need to improve their information literacy level, fully exploit the educational value and teaching advantages of information technology, promote the development process of education informatization and modernization, and help cultivate sports talents.

## Data availability statement

The original contributions presented in the study are included in the article/Supplementary material, further inquiries can be directed to the corresponding author.

## Author contributions

TH: overall paper design and writing and revising papers. HL and FX: data collection and analysis and paper writing. All authors contributed to the article and approved the submitted version.

## Funding

This research was supported by the Program of National Social Science Foundation (Grant No. 20BTY059).

## Conflict of interest

The authors declare that the research was conducted in the absence of any commercial or financial relationships that could be construed as a potential conflict of interest.

## Publisher’s note

All claims expressed in this article are solely those of the authors and do not necessarily represent those of their affiliated organizations, or those of the publisher, the editors and the reviewers. Any product that may be evaluated in this article, or claim that may be made by its manufacturer, is not guaranteed or endorsed by the publisher.

## Supplementary material

The Supplementary material for this article can be found online at: https://www.frontiersin.org/articles/10.3389/fpsyg.2023.1060257/full#supplementary-material
