# Supplementary material for: Research on the design and application of “MOOC + flipped classroom” for basketball courses in colleges and universities from the perspective of education modernization
Source: Front Psychol. 2023 Jan 25;14:1060257. doi: 10.3389/fpsyg.2023.1060257 (PMC9905832; doi:10.3389/fpsyg.2023.1060257)
Supplement: Supplementary file 1 [file Table_1.DOCX]

Supplementary Material

# Supplementary Data

## Supplementary Figures


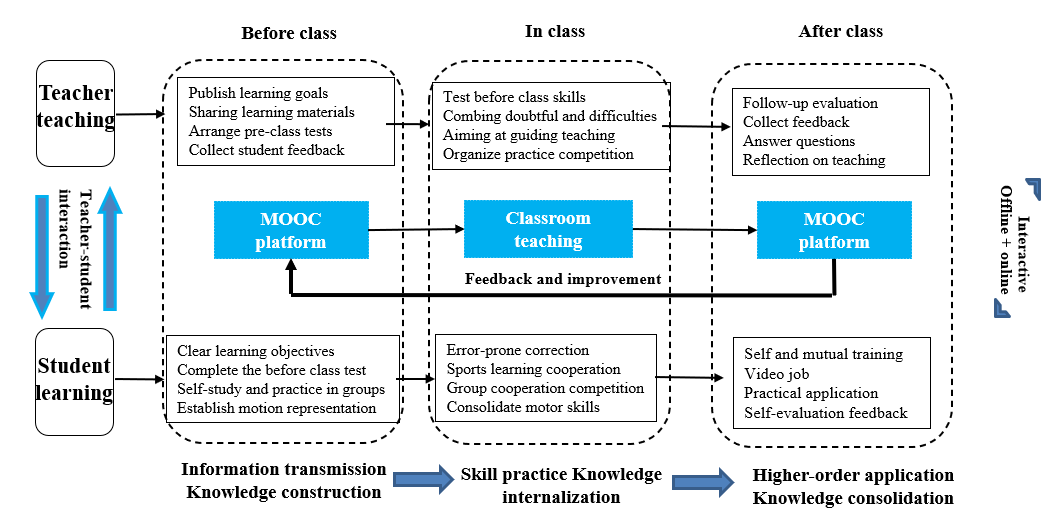


**Supplementary Figure 1.** *Teaching design of "MOOC+ flipped classroom" for basketball courses in colleges and universities*


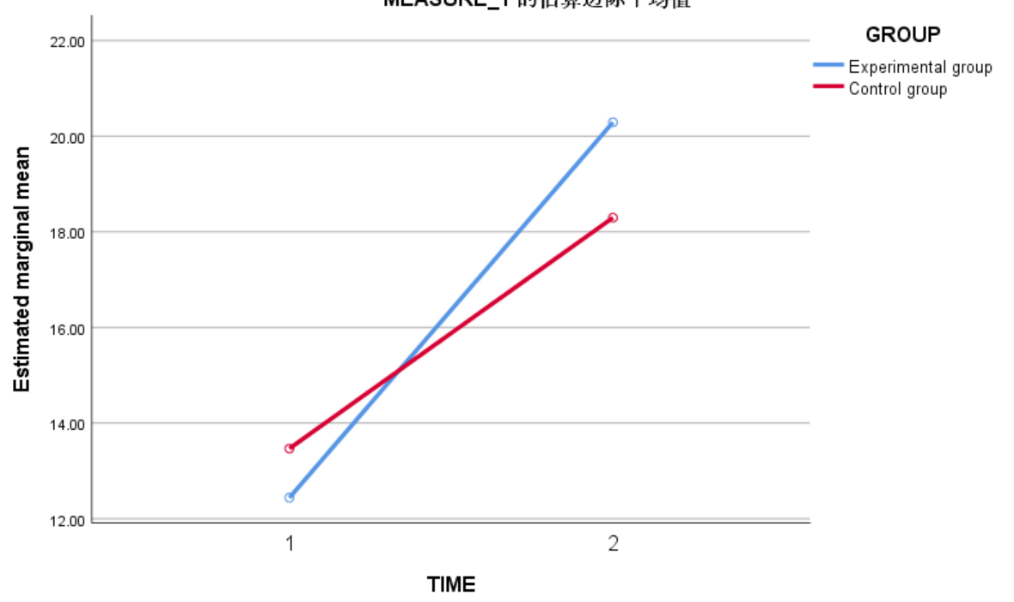


**Supplementary Figure 2.** *Schematic diagram of the changes in basketball skill level between the experimental and control groups before and after the intervention*


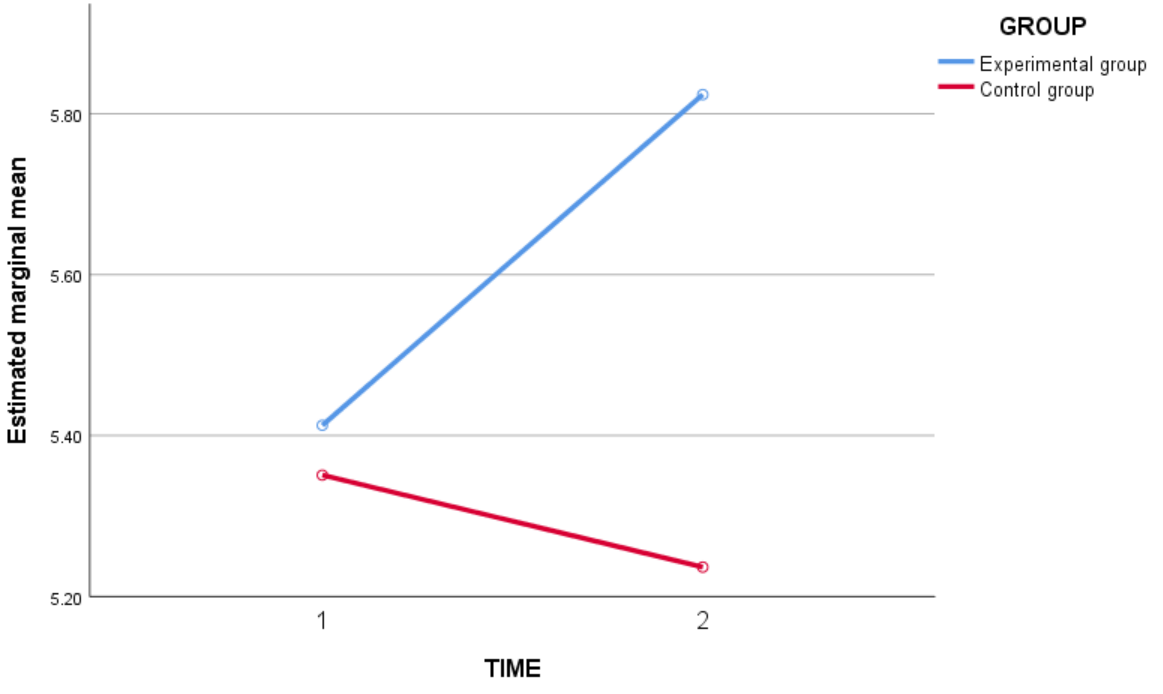


**Supplementary Figure 3.** *Schematic diagram of changes in study engagement on the experimental group and the control group before and after the intervention*


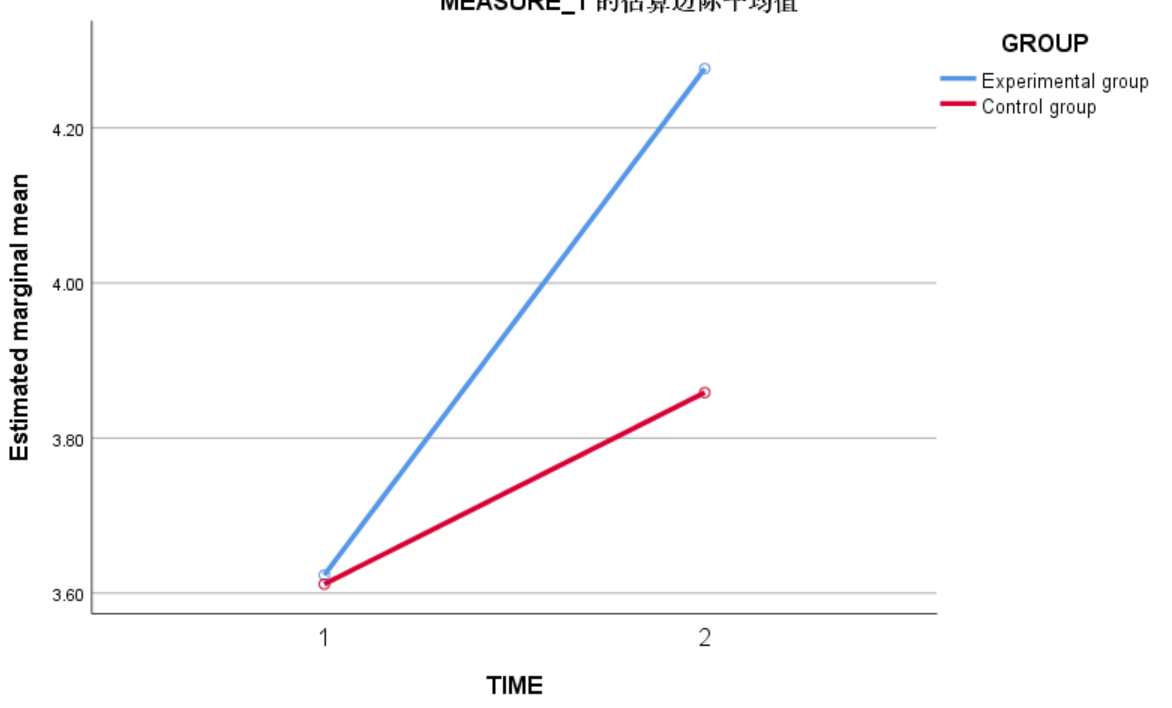


**Supplementary Figure 4.** *Schematic diagram of the changes in cooperation ability between the experimental group and the control group before and after the intervention*


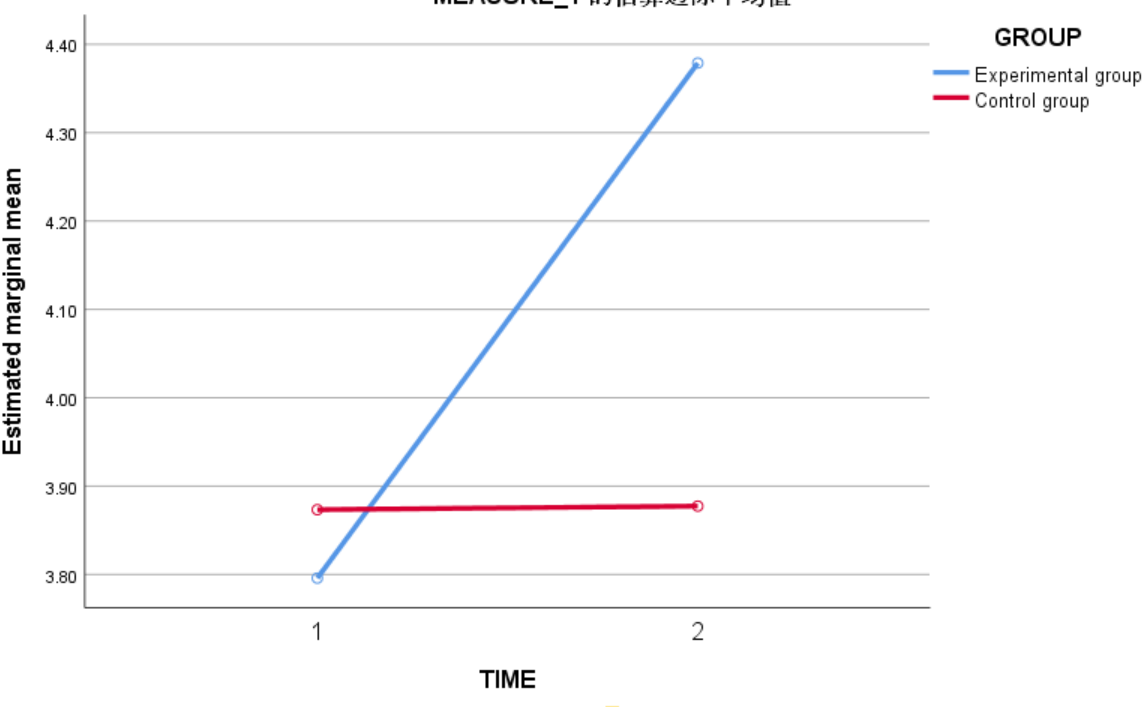


**Supplementary Figure 5.** *Schematic diagram of changes in self-regulated learning ability between the experimental group and the control group before and after the intervention*

## Supplementary Tables

**Supplementary Table 1.** *Analysis of t-test results of independent samples in the experimental group and the control group*

|  | Group (*M*± *SD*) | | *t* | *p* |
| --- | --- | --- | --- | --- |
|  | Control group (n=30) | Experimental group (n=34) |  |  |
| Basketball skill level | 13.47±4.18 | 12.44±3.81 | 1,028 | 0.308 |
| Study engagement | 5.35±1.23 | 5.41±0.95 | -0.226 | 0.822 |
| Cooperation ability | 3.61±0.50 | 3.62±0.61 | -0.082 | 0.935 |
| Self - regulated learning ability | 3.87±0.51 | 3.80±0.39 | 0.683 | 0.497 |

**Supplementary Table 2.**  *Repeated measures ANOVA results of experimental group and control group*

|  | The source | Post -test | | *F* | *p* |
| --- | --- | --- | --- | --- | --- |
|  |  | Experimental group（*M*±*SD*） | Control group（*M*±*SD*） |  |  |
| Basketball skill level | Time | 20.29±1.91 | 18.29±1.92 | 209.150 | 0.000** |
|  | Group |  |  | 0.557 | 0.458 |
|  | Time * Group |  |  | 11.878 | 0.001** |
| Study engagement | Time | 5.82±0.85 | 5.24±1.17 | 1.905 | 0.172 |
|  | Group |  |  | 1.818 | 0.182 |
|  | Time * Group |  |  | 5.970 | 0.017* |
| Cooperation ability | Time | 4.28±0.41 | 3.86±0.50 | 92.894 | 0.000** |
|  | Group |  |  | 3.668 | 0.060 |
|  | Time * Group |  |  | 15.849 | 0.000** |
| Self - regulated learning ability | Time | 4.38±0.43 | 3.92±0.54 | 13.856 | 0.000** |
|  | Group |  |  | 5.891 | 0.018* |
|  | Time * Group |  |  | 9.440 | 0.003** |

*Note.* **means p＜0.01, *means p＜0.05

**Supplementary Table 3.** *Simple effects analysis of basketball skill level group between experimental group and control group before and after the intervention*

| Time | Group | Average difference | | | Standard error | *t* | *p* |
| --- | --- | --- | --- | --- | --- | --- | --- |
| Pre -test | Control group - Experimental group | 1.026 | 0.783 | | | 1,310 | 0.193 |
| Post -test | Control group - Experimental group | -1.995 | | 0.783 | | -2,547 | 0.012* |

*Note.* *means *p*＜0.05

**Supplementary Table 4.** *Simple effects analysis of study engagement group between experimental group and control group before and after intervention*

| Time | Group | Average difference | | | Standard error | *t* | *p* |
| --- | --- | --- | --- | --- | --- | --- | --- |
| Pre -test | Control group - Experimental group | -0.062 | 0.264 | | | -0.235 | 0.815 |
| Post -test | Control group - Experimental group | -0.587 | | 0.264 | | -2,227 | 0.029* |

*Note.* *means *p*＜0.05

**Supplementary Table 5.** *Simple effects analysis of cooperation ability group between experimental group and control group before and after intervention*

| Time | Group | Average difference | | | Standard error | *t* | *p* |
| --- | --- | --- | --- | --- | --- | --- | --- |
| Pre -test | Control group - Experimental group | -0.012 | 0.128 | | | -0.090 | 0.928 |
| Post -test | Control group - Experimental group | -0.418 | | 0.128 | | -3,265 | 0.002** |

*Note.* **means *p*＜0.01
